# Supplementary material for: Identification of Genes Associated with Gut Microbiota Metabolites in Type 2 Diabetes Mellitus Complicated by Acute Cerebral Infarction Based on Bioinformatics and Mendelian Randomization
Source: Biomedicines. 2026 Oct 17;14(10):2371. doi: 10.3390/biomedicines14102371 (PMC13702449; doi:10.3390/biomedicines14102371)
Supplement: Supplementary file 1 [file biomedicines-14-02371-s001.zip › Figure S3/family.Verrucomicrobiaceae.id.4036 T2DM.pdf]

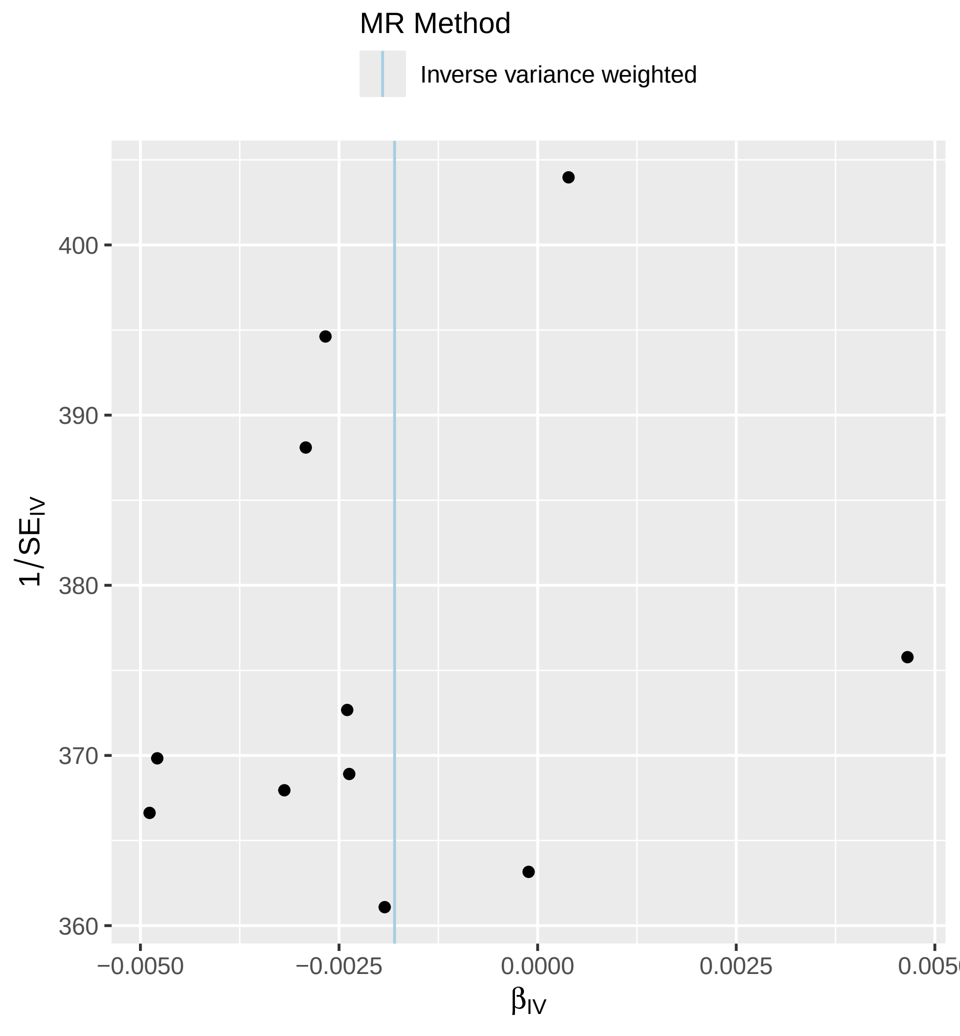
MR Method
Inverse variance weighted
400
390
380
370
360
1/SE_IV
−0.0050
−0.0025
0.0000
0.0025
0.005
β_IV
